# Supplementary material for: Determinants of patient satisfaction: Lessons from large-scale inpatient interviews in Vietnam
Source: PLoS One. 2020 Sep 18;15(9):e0239306. doi: 10.1371/journal.pone.0239306 (PMC7500619; doi:10.1371/journal.pone.0239306)
Supplement: Attachment — Submitted filename: PlosOne-Response letter - 2019-01-16.docx [file pone.0239306.s001.docx]

**In response to comments from Editor:**
**E1.** (We will number these comments consecutively in order to refer back to them as needed)

Reviewer 1 presents a clear and concise list of comments and suggestions on methodological issues, and on format and presentation. I suggest each one of Reviewer 1's points be carefully addressed. Reviewer 2 agrees with some of Reviewer 1's points, and where both reviewers agree, those points should be given very careful attention. Reviewer 1 brings up an issue that escaped my notice, which is that there are no line numbers in the submission. Please ensure line numbers are in the revised submission and if you have difficulty with the PLoS ONE submission system in producing them in the revised manuscript, please contact the production staff for assistance.

Thanks for your review. We added line numbers in the revised submission.

**E2.**
Reviewer 2 raises two important concerns. The first concern is that the manuscript is vague on the primary source of the data. I interpreted the manuscript as saying that the primary data reported in the main results are from the MSA, but Reviewer 2 interprets it as saying that the data come from several sources. Please clarify this issue. Reviewer 2's second concern, based on their experience with survey research and data collection in Vietnam, is that MSA data may be biased due to influence of data collection personnel on patient responses. Please provide a careful response to Reviewer 2 on this point. If you agree that bias in the MSA data might be an important issue, I wonder if you have enough data from other sources to perform a sensitivity analysis or check on the primary source of data.

Thank you for raising a number of important issues through this comment. This study is the secondary data analysis using the data of the Vietnam Initiative which has been independently collected from the Ministry of Health of Vietnam (MSA). We added a flow diagram of the data collection process to clarify the source of the data and involved parties. In particular, Vietnam Initiative is the principle investigator to collect the data and provide relevant policy reports to stakeholders and the public. They received a grant from Oxfam Vietnam in the project titled “Equitable Healthcare through Patient Satisfaction Index”. The funder did not have any roles in this project’s design.

**E3.**
Finally, there are two important issues regarding the statistical methodology used, and for which set of data. The manuscript reports use of data that use two different scales for two different outcome variables. The first is a patient assessment of the degrees to which their expectations were met, which can exceed 100 percent; the second is a 5 point rating scale of patient satisfaction, which is converted to a 0-1 binary scale to indicated 'unsatisfied' versus 'satisfied' (Methods section page 6). However, the Results section appears to only report the second outcome measure. This issue need to be clarified. I don't see where the results on first set of outcome measures are reported. Were the measures of degree to which patient expectations were met converted to a 0-1 binary scale as well, and reported with the other type of outcome variable? Reviewer 2 seems to say that the first set of outcomes is reported, and wonders how patient satisfaction can exceed 100 percent. But I can't find where analysis for this first set of outcome is reported, and I admit I am guessing that what you mean for this set of outcomes is patient reports of the degree to which their expectations are met (so it can exceed 100 percent). Please provide more detail on the definition of this set of outcomes, and make sure it is clear to reader which set of outcomes is reported in each section of the Results.

Thank you. We have reorganized the method section in which more detail on the definition of outcomes has been provided in a subsection *Measure*s. We also interpret the results in the appropriate subsections of the Results.

**E4.**

Second, I interpret the manuscript as saying the primary results reported use logistic regression, and ordinary least squares (OLS) was used for a sensitivity analysis that is reported in a supplementary table (Results section, page 9) . However I don't see the supplementary table with the submission. If the supplementary table is to remain unpublished, then the authors should state that. If not, please ensure that any supplementary material is included in the revised submission. In any case, the results of the OLS sensitivity analysis should be described in the main text, and I can't find where it is. Reviewer 2 interprets the manuscript as reporting some main results are from the OLS regression, and says that these results should be omitted, or the analysis changed to use only logistic regression, since logistic regression is the only correct regression method. However, all the main results seem to be from logistic regression to me. So, please clarify this issue.

Regarding the issue of logistic regression versus OLS for binary data, I don't completely agree with Reviewer 2. In some cases OLS is an acceptable regression approach as well as logistic regression, and can be more robust. All the explanatory variables used for your analysis appear to be 0-1, and you have a relatively large number of observations, so some objections to OLS don't hold. The fact that all the explanatory variables are 0-1 should keep the predicted probabilities from the estimated model between 0 and 1, for example.

The question is how to compare the results from logistic and OLS regressions, since logistic regression and OLS report the coefficients on a different scale. One method is to pick a reference slope coefficient for the logistic and OLS regressions, and find the ratio of the other coefficients to the reference coefficient for the logistic and OLS regressions, respectively. The ratios should be approximately the same for each regression. The second approach is to compare the predictions of the logistic and OLS regressions. If both methods are appropriate for your data set, both should produce similar predictions, and the correctly and incorrectly classified observations should have substantial overlap. If the two methods produce substantially different predictions, the method with the best overall predictive performance should be preferred. Also, you may want to try ordered logit regression, which preserves all the information in the 5 point scale, and avoid the need for dichotomization of patient responses. Standard errors that are robust to heteroskedasticitiy should be used for the OLS regression. A routine for ordered logit regression should be provided in major statistical packages, such as SAS and Stata. These issues are discussed at length in the text by Jeffrey Wooldridge, Econometric Analysis of Cross Section and Panel Data, MIT Press, second edition, chapters 15 and 16 (I think the information is in the first edition too, but not sure in which chapters).

Thank you for raising a number of important methodological issues through this comment. We revisited our presentation of the results and made in a number of adjustments:

(1) Measures of overall patient satisfaction score: we used 2 measures: (i) continuous measure of satisfaction score (0 to 200 percent), using the OLS model; (ii) binary indicators of satisfaction (use of a threshold to define whether patients’ expectations were metor even exceeded), using the logistic model. We did not intend to compare the results from logistic and OLS regressions in this analysis.
(2) Measures of perceptions on a specific dimension of a healthcare setting: keep the original 5 point scale, using the ordered logistic model.

This study is the secondary data analysis using the data of the Vietnam Initiative which has been independently collected from the MSA . We added a flow diagram of the data collection process to clarify the source of the data and involved parties. In particular, Vietnam Initiative is the principle investigator to collect the data and provide relevant policy reports to stakeholders and the public. They received grant from Oxfam Vietnam in the project titled “Equitable Healthcare through Patient Satisfaction Index”. The funder did not have any roles in this project’s design.

**E5.**
The authors should state what statistical package was used for the estimation (SAS, SPSS, Stata, something else?).

I also agree with both reviewers that much of the content in the manuscript is in the wrong section. For example, describing the OLS sensitivity analysis should be in the Methods section, not the Results section. Also, the descriptive statistics of the data are presented in a 'Descriptive information' section. I believe that unless there was something unexpected in the data that lead to a change in analysis, the descriptive statistics are customarily reported in the Results section.

Thank you. We provided the details of the statistical package in the newly added subsection Statistical Analysis. We have reorganized the method and results sections for a better presentation of the analysis.

**E6.**

Please ensure that your manuscript meets PLOS ONE's style requirements, including those for file naming. The PLOS ONE style templates can be found at

http://www.journals.plos.org/plosone/s/file?id=wjVg/PLOSOne_formatting_sample_main_body.pdf and http://www.journals.plos.org/plosone/s/file?id=ba62/PLOSOne_formatting_sample_title_authors_affiliations.pdf

Thank you. We used the latex template from PLOS ONE and followed your formatting guidelines.

**E7.**

2. We note that you have indicated that data from this study are available upon request. PLOS only allows data to be available upon request if there are legal or ethical restrictions on sharing data publicly. For information on unacceptable data access restrictions, please see http://journals.plos.org/plosone/s/data-availability#loc-unacceptable-data-access-restrictions.

In your revised cover letter, please address the following prompts:

a) If there are ethical or legal restrictions on sharing a de-identified data set, please explain them in detail (e.g., data contain potentially identifying or sensitive patient information) and who has imposed them (e.g., an ethics committee). Please also provide contact information for a data access committee, ethics committee, or other institutional body to which data requests may be sent.

 b) If there are no restrictions, please upload the minimal anonymized data set necessary to replicate your study findings as either Supporting Information files or to a stable, public repository and provide us with the relevant URLs, DOIs, or accession numbers. Please see http://www.bmj.com/content/340/bmj.c181.long for guidelines on how to de-identify and prepare clinical data for publication. For a list of acceptable repositories, please see <http://journals.plos.org/plosone/s/data-availability#loc-recommended-repositories>.

Thank you for raising this concern. We provided more detail on data collection and data access. We uploaded the minimal anonymized data set and Stata codes necessary to replicate our study findings to github, a public repository (<https://github.com/nguyendieuthuy/PatientSatisfaction.git>). We also provide the contact of the third party where researchers may obtain the full dataset.

3. Please upload a copy of Supporting Information S1 Table which you refer to in your text on page 16.

We removed this Supporting Information S1 Table as we decided to use the OLS results as one of the main tables.

**In response to comments from Reviewer 1:**
**R1-1.** (We will number these comments consecutively in order to refer back to them as needed)
- Format:
o There is no line number as requested by the journal. It is a bit difficult to review and cite where are the issues.

o The section Conclusion should be renamed as Discussion.

Thanks for your review. We added line numbers and renamed the Conclusion section as Discussion in the revised submission.

**R1-2.**
- Page 3, paragraph 1: Relevant literature about correlation between patient characteristics with patient satisfaction is not sufficient, the author just focused on educational level.

Thank you for your suggestion. We have added the paragraph (line 49-76) to further discuss different patient predictors of patient satisfaction.

**R1-3.**
- Page 5: this content is repeated at the methods section and should be in the methods section

Thank you. We have removed most of this paragraph (in the Introduction section) as we now discuss this content in the design and setting subsection.

**R1-4.**
- Page 7, 8:
o Providing a diagram could be helpful to understand the sampling approach and data.
o How long for the interview? Did participants receive any thing for their participation? What is the relationship between the research team and the surveyors? Did the surveyors receive training for interview?

Thank you for your suggestion. We have provided a flow diagram to visualize the sampling approach as well as more detail on the interview process and incentives. An average phone call lasted from 7 to 10 minutes to collect responses on 12 key questions and some demographics of patients. Participants did not receive any incentives. Data of our secondary data analysis study comes from Vietnam Initiative. This institute hired an independent contractor to conduct the interviews. Vietnam Initiative also provided some training for surveyors.

**R1-5.**
o What is the software used for data analysis?

Thank you for asking this question. We performed regression analysis using Stata, version 16.0. This information was added to the Statistical Analysis subsection.

**R1-6.**
- Page 9:
o The insurance rate of Vietnam should be updated as it has been increasing significantly recent years

Thanks for the review. We have updated the insurance rate of Vietnam by the end of May 2019 as 89% of the population (84 million people).

**R1-7.**
o Text in result should not repeat figures that mentioned in the tables
o Table 1: binary variables should be presented with percentages, not MEAN. I am a bit confused about the value of the over patient satisfaction level. Please explain how you have the overall patient satisfaction level: overall needs met with min/ max value are 0/200. This should state in the method section.

Thank you. We replaced the percentages for the binary variables. We explained measures of the overall patient satisfaction level in the method section. The score’s range is usually 0% to 100%. It can exceed 100% if a patient perceived that above 100% of their expectation and needs were met (exceedance of complete expectation).

**R1-8.**
- Page 10:
o From line 19: should be presented in the discussion section
- The outcomes should be compared with similar studies in Vietnam and in other countries.
- Page 15: “Female, poor, rural patients, students, and social beneficiaries tend to have higher levels of patient satisfaction in public hospitals, the primary healthcare providers in Vietnam. These findings suggest that the public healthcare system in Vietnam still meets the medical needs for relatively vulnerable patient populations” ◊ the language here is quite personal view, lack of evidence from this part of result to contribute for this statement. Those factors could affect to medical literacy. Patient expectations and patient satisfaction is then impacted by medical literacy level.

Thank you for suggesting this. We moved this discussion from page 10 to the discussion section. In the revised manuscript, the outcomes have been compared with similar studies in Vietnam and other countries (Lines 400-430). We removed the statement “These findings suggest that the public healthcare system in Vietnam still meets the medical needs for relatively vulnerable patient populations” as it is not strongly supported by the findings of this study.

**In response to comments from Reviewer 2:**
**R2-1.** (We will number these comments consecutively in order to refer back to them as needed)

1. Introduction:
- much information referenced in this part or even in other parts of the manuscript is not updated (many cited materials were published around 2000-2004)
- in page 3 (line 10-13), I was confused about a statement "Although studies on patient satisfaction have been increasing in developing countries, publications using high-quality and large-scale patient surveys are still scarce due to resource and time constraints". When reading this, I expected that I could have an opportunity to see some discussion or supporting evidence from developing countries, yet, all of a sudden, only Turkey (developed country) and Bangladesh case studies were presented. I do believe that's not coherent and convincing as we know so many studies on patients satisfaction on healthcare were conducted and reported in both advanced and less advanced nations.
- in page 3 (line 3-11 from the bottom), instead of just listing prior studies in Vietnam, it's expected to discuss some brief results from such studies (prevalence of satisfaction, associated factors, limitations) to see the existing gaps.

Thank you for raising these concerns. In the revised manuscript, we attempted to have more updated citations. We also provided more comprehensive discussion of evidence from developing countries. On page 3, we now have expanded this discussion to emphasize the existing gaps (Lines 49-76).

**R2-2.**

- page 5 (the last paragraph), interviewing 10,489 patients is huge, I just wonder how data collectors handled this: Is it just telephone- or face-to-face-based interview or combined? More descriptions provided in methods section will help readers understand.

Thank you for your suggestion. We have provided a flow diagram to visualize the sampling approach as well as more detail on the interview process and incentives. An average phone call lasted from 7 to 10 minutes to collect responses on 12 key questions and some demographics of patients. Participants did not receive any incentives. Data of our secondary data analysis study comes from Vietnam Initiative. This institute hired an independent contractor to conduct the interviews. Vietnam Initiative also provided some training for surveyors.

**R2-3.**

2. Materials and Methods:

- The info/descriptions in this part are really mixed up, it's not really clear. I suggest that authors start with describing the design and setting, then methods, data collection and analysis. It's not logical to present the procedure of regression at the beginning of this section.

Thank you for your suggestion. We have reorganized the methods sections for a better presentation of the analysis as suggested.

**R2-4.**
- There are many pieces of information that confused readers such as pilot, why is what is pilot and why is pilot as I thought the pilot is just something the researchers did before the official study. The process of data collection is not well described such as it was said that the surveys were conducted by the Medical Services Administration (MSA) under VMOH (Vietnam Ministry of Health), Vietnam Initiative of Indiana University and Oxfam Vietnam. I think collecting data in this way could introduced a lot of bias to data. I have been working in Vietnam for more than 20 years (with focusing on research and teaching on health system, health policy and management and with a lot of interactions with policy makers in VMOH at the national and sub-national meetings and conferences; most of people raised the issue of bias about the way data were collected using the hospital system itself (such as nurses and/or doctors wearing blue/uniform clothes can not give patients freedom to report their true responses or even with data collected by MSA may not be a good way to produce valid data because MSA is just one of divisions of MOH, patients and people would be afraid of not reporting positive results in clients/satisfaction surveys because of the intervention or presence of MOH officials or staffs. Over the past years, VMOH and hospital leaders in Vietnam suggested that there be a national independent agent/organization totally independently of VMOH and the healthcare system to conduct the survey on patients satisfaction, but so far it's not feasible due to public budget limitation. In your current surveys, even you had Vietnam Initiative of Indiana Uni and Oxfarm Vietnam involved, but the main contact was still MSA of MOH.

Thank you for raising these important concerns. Data of our secondary data analysis study comes from Vietnam Initiative. We have provided a flow diagram (Fig 1) to visualize the sampling approach as well as more detail on the interview process and incentives. We removed “pilot” as we agree that this word is confusing and not accurate. Vietnam Initiative hired an independent contractor to conduct interviews. Patients or their representatives (who accompanied the patients during their hospital treatment) were followed up with shortly after being discharged (2 to 5 months) and asked about their experiences and assessments of the overall satisfaction and specific attributes of their hospital services. The sponsor of the project “Equitable Healthcare through Patient Satisfaction Index”, Oxfam Vietnam, did not have any role in the survey design and data collection process.

**R2-5.**
- Also, how to have a patient's contact info is an issue. Did every single hospital asked for patients' informed consent to provide to MSA (MOH) so the surveys were conducted or without any informed consents?If this process was actually done? How was it undertaken?...

Although Vietnam Initiative received the administrative list of patients from voluntarily participating hospitals, the surveyors gathered contacted patients before proceeding their interview. We have provided more detail on the process in the data source subsection.

**R2-6.**

- What was the fund of the study: Authors mentioned that there was not fund for this research. However, I guessed that although there was no direct fund for incentives for patients, MOH(VMA) and/or Vietnam Initiative of Indiana Uni and Oxfarm Vietnam may actually provide some financial support for some basis logistic stuff such as contact, travel, data analysis and management, etc) because this study was really costly with alot of surveys and admin processes?

This study is the secondary data analysis. There was no funding for this research. In the project “Equitable Healthcare through Patient Satisfaction Index”, the PI (Vietnam Initiative) had previously obtained grant funding from Oxfam Vietnam. Participants (patients) did not receive any incentives. We provided more detail in the revised manuscript.

**R2-7.**

- May be there was a mistake in reporting Cronbach's alpha, as I know that this coefficient is not analysed for each item, but it is done for a group of items (say at least two items or more). If so, please correct this across the methods section as well as the whole paper.

We agree with this comment. We removed this coefficient from the manuscript.

**R2-8.**

- When authors said 95% and 89% in 2017 and 2018 participated, it's not necessary to remention "Remarkably, only 5% and 10.6% of all contacted patients refused to participate to the 2017 interviews and the 2018 interviews, respectively".

Thank you. We removed this sentence to avoid repetition.

**R2-9.**
- As this study was a focus on inpatients, may be authors should use inpatients in the title because the experience and satisfaction between inpatients and outpatients are quite different
- 16% of all patients perceived over 100% of their needs met seems impossible?

Thank you. We adjusted our title as the following: “Determinants of Patient Satisfaction: Lessons from Large-Scale Inpatient Interviews in Vietnam”.

We explained measures of the overall patient satisfaction level in the method section. The score’s range is usually 0% to 100%. It can exceed 100% if a patient perceived that above 100% of their expectation and needs were met (exceedance of complete expectation).

**R2-10.**
3. Results:
- As this study, logistic regression was applied for inferential analysis, the OLS is not appropriate because it is only used for linear regression, while logistic regression works with the method of maximum likelihood estimation (MLE).

Thank you for raising this issue. We revisited our presentation of the results and made a number of adjustments:

(1) Measures of overall patient satisfaction score: we used 2 measures: (i) continuous measure of satisfaction score (0 to 200 percent), using the OLS model; (ii) binary indicators of satisfaction (use of a threshold to define whether patients’ expectations were metor even exceeded), using the logistic model.
(2) Measures of perceptions on a specific dimension of a healthcare setting: keep the original 5 point scale, using the ordered logistic model.

**R2-11.**
- As there is no discussion section, it's really difficult to distinguish between results and discussion as the authors mixed them up in one section "results'

Thank you. We renamed the conclusion section to Discussion section. The discussion of the results has been moved to the Discussion section.

**R2-12.**
- As this study was Vietnam-based context, it's also better to compare with other studies in Vietnam (I could not see much this comparison in this section)

- There is a quite interesting result "female patients, patients older than 50 years of age, rural patients, and those with only primary level education expressed higher scores for medical staff", please explain why or provide some possible assumptions for this as this results was just put forward in the text.

Thank you for this suggestion. In the revised manuscript, the outcomes have been compared with similar studies in Vietnam and other countries (Lines 400-430).

**R2-13.**
4. Conclusions:
- The first sentence was too lengthy
- The result or conclusion "Female, poor, rural patients, students, and social beneficiaries tend to have higher levels of patient satisfaction in public hospitals, the primary healthcare providers in Vietnam." could be misunderstood if no explanations are provided; perhaps these groups have low expectations and demands.

We removed the statement “These findings suggest that the public healthcare system in Vietnam still meets the medical needs for relatively vulnerable patient populations” as it is not strongly supported by the findings of this study.

**R2-14.**
5. Limitations:
- Two other limitations are 1) cross-sectional design can not determine causality; 2) the nature of surveys conducted by MOH introduces some bias.
- Another limitation could be due to the nature of data collection conducted by MOH/MSA.

Thank you for your suggestion. We added the first limitation to our Discussion section. As discussed earlier, the data collection was independently conducted by a contracter, Development and Policies Research Center (DEPOCEN). This contracter is independent from Vietnam Initiative, funder (Oxfam), and MOH/MSA.

**R2-15.**

6. References:
- Not updated because just 6 among 24 items have been published within 5 recent years.

Thank you for raising this issue. In the revised manuscript, we attempted to have more recent citations.
