# Supplementary material for: Determinants of patient satisfaction: Lessons from large-scale inpatient interviews in Vietnam
Source: PLoS One. 2020 Sep 18;15(9):e0239306. doi: 10.1371/journal.pone.0239306 (PMC7500619; doi:10.1371/journal.pone.0239306)
Supplement: Attachment — Submitted filename: PlosOne-Response letter - 2020-07-05.docx [file pone.0239306.s002.docx]

**In response to comments from Editor:**
*“While the manuscript is much improved, Reviewer 1 is of the view that additional revisions are needed to address his/her comments. I look forward to seeing a revised version of the manuscript along with a point by point response.”*

Thank you for your continued consideration of our manuscript. We submit here a revised version of our article, which addresses the concerns raised by Reviewer 1. We also include a point-by-point response to each of the comments below.

**In response to comments from Reviewer 1:**

**R1-1.** (We will number these comments consecutively in order to refer back to them as needed)

*“Objectives of the study were not presented clearly and concise (line 149-169).”*

Thank you. We have revised the introduction to present the objectives in a more concise fashion in lines 48-69 and lines 97-103 (the version with track changes). One example is the following:

“This study examines factors associated with inpatient satisfaction levels in Vietnam's public hospitals during 2017-2018, using nationwide, large-scale interview data from randomized and voluntary responses to a survey provided to 69 large and public hospitals. We look at how patient satisfaction levels respond to service-specific characteristics (length of stay and extra-paid services), patients' financial conditions (employment status, health insurance, and income level), and demographic factors (age, gender, race, location and level of education). We further evaluate the associations of these factors with different salient aspects of their healthcare service experience: hospital staff, hospital facilities, and treatment costs.”

**R1-2. “***The method section was not well organized and scientific. It is very nice that the authors added a flow diagram to help visualize the sampling approach. If the authors put a bit more notice on presenting the study design, data collection and also describing major variables with their value, it would be easier for readers to follow.”*

Thank you for this comment. We have made a number of edits in the method section to improve the presentations of our study design and measures. The changes are color-coded in blue (lines 187-190, 236-341, and 266-271).

R1-3. “*Such as the overall patient satisfaction level, in line 261 -265 p10, was stated that: “The score' range is usually 0% to 100%. It can exceed 100% if a patient perceived that above 100% of their expectations and needs were met (exceedance of complete expectation).” But in Table 1 p34, the range is [0-200]. Maybe I missed something…”* The authors should consider about how to report the dependent variables as the previous comments and then present the results following the major objectives order. I feel confused and have to read several times to understand.

Thank you for raising this point. We made some edits to provide some details of this variable in lines 316 –323, following:

“The range of the observed overall patient satisfaction level is between 0% and 200%, a higher score indicates a higher level of patient satisfaction (first row). The median value and average score are 85% and 82.9%, respectively. The reported score' range is usually 0% to 100%, only 10 patients of 10,143 respondents reported a score that exceeds 100% (7 respondents with a 110% and 3 respondents with a 200% score). It implies that these patients perceived that above 100% of their expectation and needs were met (exceedance of complete expectation).”
